# Editorial: Prenatal Beginnings for Better Health

**DOI:** 10.3389/fphar.2018.00457

**Published:** 2018-05-04

**Authors:** Edward Kim, Maged Costantine, Ahmet A. Baschat, Irina Burd

**Affiliations:** ^1^Integrated Research Center for Fetal Medicine, Maternal Fetal Medicine, Department of Gynecology and Obstetrics, School of Medicine, Johns Hopkins University, Baltimore, MD, United States; ^2^Maternal Fetal Medicine, Department of Obstetrics and Gynecology, University of Texas Medical Branch at Galveston, Galveston, TX, United States; ^3^Department of Gynecology and Obstetrics, Center for Fetal Therapy, School of Medicine, Johns Hopkins University, Baltimore, MD, United States

**Keywords:** prenatal, *in utero*, origins of health, origins of desease, perinatology

Prenatal care of pregnant women traditionally focuses on potential emergence of complications in the current pregnancy. Adverse pregnancy outcomes such as preterm labor, premature preterm rupture of membrane, gestational diabetes mellitus, and preeclampsia are known to have long-term health implications for the mother and child. Recent research developments of the specific downstream health risks of pregnancy complications hold promise for preventive measures including progesterone therapy, cerclage, acetylsalicylic acid therapy that can potentially avert the long-term health impacts. Furthermore, the drive to develop more accurate and non-invasive ways to detect pregnancies at risk of maternal and neonatal morbidities has led to investigation of biomarkers. With this trend in mind, we established this Research Topic, hosted by Obstetric and Pediatric Pharmacology, a joint division of Frontiers in Pediatrics and Frontiers in Pharmacology. Our aim is to review submissions made to this special Topic, which encompasses some of the latest discovery in biomarkers, molecular pathways of pregnancy complications and long-term health consequents of adverse pregnancy outcomes on the offspring. This Topic brings together 11 articles, with broad scope, in a novel multidisciplinary collaboration among obstetrics & gynecology, pharmacology, neurology, and pediatrics. These articles are organized around critical periods in pregnancy: pre-conception, antepartum, peripartum, and immediately postpartum.

In the pre-conception period, a woman's baseline health may foreshadow short and long-term health consequences for the offspring, as outlined by Arabin and Baschat. Women with preexisting hypertensive and metabolic risk profiles are more vulnerable to development of preeclampsia. Offspring born to mothers affected by preeclampsia, in turn, are at increased risk for hypertension, cerebrovascular accident, cognitive delay and depression, with the risk significantly increased for those affected by preterm preeclampsia. Placental stressors, inadequate delivery of nutrients *in utero* due to famine, or maternal stress due to external stressors are associated with low birth weight in the offspring, which is further linked with increased risk of cardiovascular disease, dyslipidemia, and psychiatric disorders.

On the other hand, maternal dietary supplementation has been shown to promote fetal wellbeing. One of the articles in the Topic by Mozurkewich et al. suggests possible benefit of fatty acid supplementation. In this study, women on dietary supplementation containing omega-3 fatty acid docosahexaenoic acid (DHA) and eicosapentaenoic acid (EPA) had significantly high levels of metabolites of DHA and EPA in the umbilical cord blood, potentially important for reduction of preterm births and risk for infant admission to neonatal intensive care unit. While no conclusion can be drawn from this single study, it does suggest that further investigation into maternal dietary consumptions may prove beneficial for determining ways to promote fetal health.

In the antepartum period, early *in utero* diagnosis of disease is crucial in terms of parental counseling and planning fetal interventions. Jelin et al. calls attention to skeletal dysplasias and development of molecular diagnosis, bone marrow transplant, and gene therapy *in utero*. Even skeletal diseases with significant comorbidity such as osteogenesis imperfecta may theoretically be amenable for novel treatments such as stem cell and bone marrow transplantation *in utero*. Such invasive *in utero* interventions may require simultaneous development of fetal anesthesia. Smith et al. validated remifentanil as a reasonable anesthetic agent frequently used in fetal interventions.

Shifting focus to another vital part of pregnancy, the placenta, Gumina and Su investigated the possible role of placental endothelial progenitor cells (EPCs). They suggest that EPCs, which had previously been implicated in vasculogenesis and angiogenesis, may have a role in vasculaturerelated pregnancy complications such as preeclampsia and fetal growth restriction. They highlight experiments where altered balance between EPCs of various angiogenic potential was seen in cord blood of infants affected by preeclampsia and fetal growth restrict ion. Thus, they cautiously posit EPCs may play an important role in pathogenesis of these pregnancy complications and may serve as possible targets for intervention.

Labor, the key event in the peripartum period, is intricately orchestrated by the mother and fetus. However, the precise signal exchange initiating labor is not yet fully understood. Sheller-Miller et al. hypothesize that the fetus may signal for onset of labor via exosomes, specialized intracellular signaling vesicles, in a murine model. Even less is known about pathogenesis of preterm labor. Preterm labor, delivery prior to 37 weeks' gestation is the leading cause of mortality among infants otherwise with no congenital anomalies. Infants born after preterm labor are at an increased risk of long-term intellectual and physical disabilities compared with term neonates Manuck. Manuck reviews current management of preterm labor—intramuscular progesterone for prevention, and treatment with indomethacin—and calls for further investigation into pathogenesis of preterm labor. Zhao et al. posit that ubiquitin-proteasomecollagen (CUP) pathway is implicated in molecular pathogenesis of preterm labor Johnson et al. They discovered that certain messenger RNAs associated with the CUP pathway were differently expressed in placentas and fetal membranes in women who had preterm labor or preterm premature rupture of membrane (PPROM). Their research represents one of the first steps in elucidating some of the molecular mechanisms of preterm labor and PPROM. Understanding these mechanisms may help develop targeted therapy to prevent and treat these conditions.

In the immediate postnatal period, early recognition of neonates with encephalopathy could significantly improve prognosis. Lei et al. demonstrate that neuronal nitric oxide synthase (NOS1), a marker for oxidative stress, was increased in umbilical cord blood of neonates affected by encephalopathy. They speculate that NOS1 may be a viable biomarker for early identification of neonatal encephalopathy and perinatal brain injury. An article by Graham et al. feature not only NOS1, but other biomarkers being studied that could be used as a panel to supplement existing tools to evaluate encephalopathy in neonates. Refinement of neonatal encephalopathy evaluation methods may help identify neonates who would benefit from interventions such as controlled hypothermia and cost associated with management of these neonates.

We hope that this compilation of articles highlighting the latest research in obstetrics and pediatric pharmacology will be of interest to the readers and will inspire more research in this exciting multidisciplinary approach to perinatal care.

## Author contributions

All authors listed have made a substantial, direct and intellectual contribution to the work, and approved it for publication.

### Conflict of interest statement

The authors declare that the research was conducted in the absence of any commercial or financial relationships that could be construed as a potential conflict of interest.

